# Management of Sudden Sensorineural Hearing Loss among General Practitioners in the United Kingdom: A Cross-Sectional Survey Study

**DOI:** 10.1055/a-2804-6442

**Published:** 2026-09-15

**Authors:** Muhammad Umar Farooq, Priya Sunil Patel, Gursahib Singh Bal, Faisal Javed

**Affiliations:** 1 Department of Ear, Nose and Throat Surgery, University Hospitals Birmingham NHS Trust, Birmingham, United Kingdom

**Keywords:** sudden sensorineural hearing loss, hearing loss, primary care, general practice, otolaryngology, clinical guidelines

## Abstract

**Objective:**

To assess how general practitioners (GPs) in the United Kingdom recognise, investigate, and manage sudden sensorineural hearing loss (SSNHL), and to determine the extent to which their practice aligns with national guidance.

**Methods:**

A cross-sectional online survey was distributed between January and July 2025 to fully qualified GPs across the West Midlands. The questionnaire explored definitions used, diagnostic methods, management strategies, and familiarity with NICE and ENT UK recommendations.

**Results:**

Fifty GPs completed the survey. Although 84% attempted to distinguish conductive from sensorineural loss, only 56% regularly used tuning-fork tests. Urgent ENT referral was the most common initial step (68%), while only 18% initiated pharmacological therapy and 36% prescribed corticosteroids. Fewer than half were familiar with guideline content, yet 96% expressed a wish for additional training.

**Conclusion:**

SSNHL remains inconsistently recognized and managed in UK primary care. Improving GP confidence through targeted ENT education and streamlined referral pathways could substantially enhance early treatment and hearing outcomes.

## Introduction

Sudden sensorineural hearing loss (SSNHL) is defined as a rapid onset of at least 30 dB loss across three contiguous frequencies within 72 hours and represents an ontological emergency. 1 2 The annual incidence in the United Kingdom (UK) is estimated to range from 5 to 27 per 100,000 people, corresponding to approximately 3,000 to 18,000 new cases each year. 3 4 Timely recognition and corticosteroid therapy—ideally within two weeks—offer the best chance of recovery. 5

The actual pathogenesis of SSNHL remains largely debated; however, certain mechanisms have been proposed, including viral labyrinthitis, micro-vascular compromise, and autoimmune inner-ear disease. 6 7 In the UK, current National Institute for Health and Care Excellence (NICE) (NG98, updated 2023) and Ear, Nose and Throat UK (ENT UK) (updated 2024) guidance emphasizes rapid differentiation of conductive from sensorineural loss using tuning-fork tests, urgent referral within 24 hours, and early steroid administration. 8 9

Despite such clear direction, variation in primary-care management persists. Several audits have reported that more than 40% of patients are referred beyond the recommended window, and fewer than 60% of general practitioners (GPs) feel confident performing bedside tests. 10 11 12 In primary care, SSNHL is often misdiagnosed or diagnosed late as symptoms like ear fullness or sudden hearing loss are frequently mistaken for benign conditions such as wax impaction or upper respiratory infection. The lack of baseline audiometry further complicates diagnosis, and formal testing is rarely available in primary care. 13 14 Although otoscopy and tuning fork tests (Weber and Rinne) can help distinguish sensorineural from conductive loss, they are inconsistently performed and of variable accuracy. As pure tone audiometry remains the gold standard, prompt ENT referral is essential for confirmation and timely management. 15

As GPs are the initial contact for most adults with new hearing loss, adherence to evidence-based referral pathways is vital to prevent irreversible cochlear injury. This study aimed to evaluate current GP awareness, diagnostic behaviour, and management of SSNHL in the West Midlands and to identify modifiable barriers to timely care.

## Methods

A descriptive cross-sectional survey was conducted from January to July 2025 across NHS-affiliated practices in the West Midlands. Eligible participants were fully qualified GPs; trainees were excluded. Recruitment occurred via flyers displayed in surgeries and email invitations distributed through primary-care networks. Participation was voluntary and uncompensated.

The 17-item questionnaire explored demographics, understanding of SSNHL definitions, diagnostic approaches (including tuning-fork and audiogram use), management choices, and knowledge of NICE and ENT-UK guidelines. Data were collected anonymously via a secure web platform and analysed using Microsoft Excel to produce descriptive statistics. Because no identifiable information was gathered and participation was voluntary, formal research-ethics approval was not required.

## Results

### Participant Characteristics

Fifty GPs completed the survey. Their experience ranged from fewer than five years to more than twenty years; the largest group (36%) had 5–10 years of practice. Most participants were based in Birmingham and Solihull (48%), followed by Staffordshire and Shropshire (30%). 92% had encountered fewer than five patients with acute unilateral hearing loss in the preceding six months.

### Diagnostic Behaviour

Definitions of SSNHL differed widely: 36% defined onset as ≤ 72 hours; 4% ≤ 48 hours; 46% ≤ 24 hours; and 12% ≤ 7 days. Although 84% attempted to distinguish conductive from sensorineural loss, only 66% used tuning-fork tests and 58% were uncomfortable interpreting audiograms. 70% performed no investigations at initial presentation; 24% arranged audiology, 4% ordered MRI, and 2% requested blood tests.

### Management and Referral

Most GPs (68%) referred urgently to ENT, while 18% initiated pharmacological therapy and 14% directed patients to Accident & Emergency. Among prescribers, 36% of those who initiated pharmacological therapy. 52% reported familiarity with referral pathways, but only 44% were comfortable accessing guidelines. Nearly all respondents (96%) desired further SSNHL training.

## Statistical Analysis and Results

Inferential statistical analyses were conducted to explore key associations of clinical relevance:

1. Years of GP experience and use of tuning-fork tests
2. Familiarity with SSNHL guidelines and likelihood of prescribing corticosteroids
3. Accurate definition of SSNHL (≤72 hours) and likelihood of urgent ENT referral.

Chi-square (χ ^2^ ) or Fisher's exact tests were used, with odds ratios (OR) and 95 % confidence intervals (CI) calculated to estimate effect size. A two-tailed p < 0.05 was considered statistically significant.

Among 50 participating GPs, 33 (66%) reported using tuning-fork tests to differentiate conductive from sensorineural hearing loss. There was no statistically significant association between years in practice and tuning-fork use (χ ^2^ [4] = 5.94, p = 0.20), suggesting that diagnostic approach was consistent across experience levels.

Familiarity with SSNHL guidelines was strongly associated with evidence-based prescribing. Of 26 GPs familiar with referral pathways, 14 (53.8%) prescribed corticosteroids compared with 4 of 24 (16.7%) among those unfamiliar. This difference was statistically significant (OR = 5.83, 95% CI 1.56–22.0; p ≈ 0.006), indicating that awareness of guidelines markedly increases the likelihood of initiating steroid therapy.

When defining SSNHL, 44 GPs (88%) selected ≤ 72 hours (including options ≤ 24 hours and ≤ 48 hours which all fall) in accordance with NICE and ENT UK guidance. Of these, 41 (93.2%) made urgent ENT referrals compared with 4 of 6 (66.7%) of those using longer definitions. Although not statistically significant (OR = 6.83, 95% CI: 0.9 to >50; p = 0.07), this trend suggests that accurate understanding of SSNHL criteria positively influences timely referral.

## Discussion

This cross-sectional survey demonstrates important gaps between recommended best practice and real-world general practice management of SSNHL in the United Kingdom. Although most GPs recognised acute unilateral hearing loss as an urgent symptom, several essential aspects of guideline-concordant care were inconsistently implemented.

The use of tuning-fork tests was limited: only 33 of 50 GPs (66%) routinely performed them. This falls short of NICE and ENT-UK expectations, which emphasise bedside differentiation of conductive from sensorineural loss where audiometry is unavailable. 2 4 8 9 Comparable findings have been reported in Canada and Saudi Arabia, with usage rates between 49% and 52%. 14 15 In this study, no significant association was found between years in practice and tuning-fork use, indicating that underuse is not isolated to less experienced clinicians. This trend may reflect evolving training priorities and declining emphasis on ENT examination skills in the primary care curriculum. 16 17

While the majority (88%) of GPs defined SSNHL as hearing loss developing within 72 hours, 12% used broader definitions (up to 7–14 days). Accurate definition correlated with improved referral behaviour: GPs using the ≤72-hour criterion were over six times more likely to make an urgent ENT referral (OR = 6.83, 95% CI 0.9- > 50; p ≈ 0.07). Although not statistically significant, this relationship underscores the impact of clinical awareness on patient pathways and the importance of consistent diagnostic language. Misclassification risks delayed treatment and poorer outcomes.

Only 36% of GPs initiated corticosteroid therapy when SSNHL was suspected, despite strong evidence supporting early treatment. 1 5 18 Familiarity with referral pathways significantly increased the likelihood of prescribing steroids (OR = 5.83, 95% CI 1.56–22.0; p ≈ 0.006), suggesting that knowledge dissemination directly influences therapeutic decision-making. This aligns with international evidence demonstrating that guideline familiarity is the strongest predictor of evidence-based SSNHL management. 14 15 20

Encouragingly, 96% of respondents expressed a desire for additional SSNHL training, with 88% willing to attend ENT-led teaching. This enthusiasm highlights that practice gaps are rooted more in training and system constraints than in clinical disengagement. Educational interventions—such as concise online CPD modules, practical tuning-fork workshops, and integration of SSNHL prompts into electronic health record systems—could improve recognition and expedite management.

Delayed identification and referral remain major barriers to recovery. Previous UK audits found that over 40% of patients reached ENT services beyond two weeks after symptom onset, often following initial misdiagnosis as otitis media or wax impaction. 18 21 Enhancing diagnostic training and embedding decision-support tools in primary-care software could substantially reduce these delays.

Recent research supports intratympanic corticosteroid injection as both first-line and salvage therapy. 22 23 Adjunctive hyperbaric oxygen therapy (HBOT) also shows modest benefit. 24 Meanwhile, artificial-intelligence algorithms are being developed to interpret audiometric data and flag potential SSNHL cases for rapid referral. 25 Tele-ENT consultation pathways, now piloted across Canada and Europe, have demonstrated shorter waiting times and improved treatment adherence. 16 17 Implementing similar digital triage systems within the National Health Service (NHS) could strengthen early-intervention rates and reduce regional inequities.

## Conclusion

UK GPs exhibit partial adherence to evidence-based SSNHL management. Limited familiarity with guidelines, inconsistent use of tuning-fork tests, and variable prescribing behaviours contribute to delays that may compromise hearing outcomes. Strengthening undergraduate and continuing professional ENT education, combined with electronic referral prompts and tele-ENT access, could markedly improve diagnostic accuracy and timely treatment.
